# Economic Burden of Otitis Media Globally and an Overview of the Current Scenario to Alleviate the Disease Burden: A Systematic Review

**DOI:** 10.1055/s-0043-1767802

**Published:** 2024-07-05

**Authors:** Ragini Bhatia, Anil Chauhan, Monika Rana, Kulbir Kaur, Pranita Pradhan, Meenu Singh

**Affiliations:** 1 Department of Pediatrics, Postgraduate Institute of Medical Education and Research, Chandigarh, India; 2 Department of Telemedicine, Postgraduate Institute of Medical Education and Research, Chandigarh, India; 3 Advanced Pediatric Center, Postgraduate Institute of Medical Education and Research, Chandigarh, India

**Keywords:** otitis media, direct costs, indirect costs, economic burden

## Abstract

**Introduction** The prevalence of otitis media (OM) is substantial all over the world. Epidemiological data related to the economic burden of OM globally is minimal. The present systematic review was undertaken to estimate the economic burden of this disease in various parts of the world.

**Objectives** An extensive literature search was done using PRISMA guidelines to identify relevant studies that estimated the economic burden of OM in monetary terms. The databases searched were PubMed Central, Ovid, and Embase. The cost estimation was done for one specific year and then compared considering the inflation rate.

**Data Synthesis** The literature search led to the inclusion of 10 studies. The studies evaluated direct and indirect costs in monetary terms. Direct costs (health system and patient perspective) ranged from USD (United States Dollar) 122.64 (Netherlands) to USD 633.6 (USA) per episode of OM. Looking at only the patient perspective, the costs ranged from USD 19.32 (Oman) to USD 80.5 (Saudi Arabia). The total costs (direct and indirect) ranged from USD 232.7 to USD 977 (UK) per episode of OM. The economic burden per year was highest in the USA (USD 5 billion). The incidence of OM episodes was found more in children < 5 years old. Introduction of pneumococcal conjugate vaccines decreased the incidence in children and now the prevalence in adults is of concern.

**Conclusion** The economic burden of OM is relatively high globally and addressing this public health burden is important. Approaches for the prevention, diagnosis, and treatment should be undertaken by the health system to alleviate this disease burden.

## Introduction

Otitis media (OM) is the infection of the middle ear with common symptoms of pain, irritability, and fever. Otitis media can be classified regarding: 1)Duration – acute OM (AOM) and chronic OM(COM); 2) Nature of discharge – suppurative and nonsuppurative OM ;3) OM with effusion (OME); 4) Organism causing OM – bacterial OM and specific OM. 1 The systematic review done by Montasa et al. demonstrated that the worldwide AOM incidence rate is 10.85%, which adds 709 million cases each year; out of which more than half occur in children aged < 5 years old. 2 The incidence rate of chronic suppurative OM (CSOM) is 4.76%, leading to 31 million cases, with 22.6% of cases occurring annually in children < 5 years old. A major part of the disease burden is borne by the developing nations. The risk factors of ear ailments are majorly sociodemographic factors that play an important role. 3 Acute otitis media can occur at any age, but it is most common in the age group of 6 to 24 months. 4 Otitis media is associated with life-threatening complications, both intracranial and extracranial. 5 Suppurative OM can result in perforation of tympanic membrane (TM), meningitis, brain abscess, mastoiditis, hearing loss, and other complications. 6 In 2013, the evidence-based guidelines for the diagnosis and management of AOM were updated by the American Academy of Pediatrics, accentuating the role of clear visualization of the TM in the diagnosis of ear infections. 7 Antibiotics are more frequently prescribed for AOM than for any other illness of childhood. 8 Otitis media is clinically diagnosed through a physical exam called otoscopy along with the history of the patient with presenting signs and symptoms. 9

Globally, AOM has a high economic burden regarding associated direct and indirect costs. 10 11 Demographic and public health data on the incidence and cost of ailment per OM episode are significant from the point of view of understanding both the public health burden as well as the probable economic impact of this disease. Evidence-based decisions can be made by policy makers with information of the economic burden related to the disease. Therefore, these statistics are necessary to mentor public health interventions focusing on reducing the financial burden of all forms of OM, thus providing patients with better care. 12 Direct costs are associated to the resources used of the health systems and out of pocket expenditures of the patient and caregivers. Indirect or the opportunity costs assess the time that the patients spend during the disease resulting in monetary loss (transfer, wait and recovery) and are related to the wages or salary loss. 13 Also, direct costs include the measuring of intangible costs, such as quality of life (QoL) deterioration and difficulty in social communication. 14 Indirect costs are related to the losses incurred as a result of the impact of the disease. 15 The three perspectives that are mandated in economic evaluation studies are the perspective of the health system, the patient/family, and that of the society. 16 A societal perspective considers economic evaluation regarding to everyone, that is: patients, caregivers, payee, or the health system.

Health-related quality of life is a multifactorial construct that covers four dimensions: physical wellbeing, mental health, functional impairment, and interpersonal relationships. 17 These dimensions are to be captured with specific instruments having items that take into consideration the perspective of the patient or of the caregiver for themselves or as a proxy for the minor child. Subjective outcomes are of foremost importance as compared with the objective outcomes. Initially, subjective outcomes related to hearing loss were evaluated by researchers but, lately, consequences related to various forms of OM are being understood. The variable spectrum of effects that OM has on the everyday life of children and caregivers has resulted in the development of specific instruments in the form of questionnaires. The various instruments in the form of questionnaire that can be used to assess the quality of life of children with otitis media are:1) OM6; 2) Child Health Questionnaire Parent form; 3) QoL Disruption Scale; 4)Pediatric Quality of Life Inventory; 5)Health Utility Index Mark 3; 6) OM8-30; 7) Chronic OM outcome Test-15; 8) EuroQol five dimensions questionnaire. The OM-6 questionnaire is the most applied instrument to document the quality of life of the child. ^17^ A systematic review done by Homøe et al. 18 reviewed 15 studies depicting that QoL is a highly subjective measure related to patient preferences. A systematic review done by Chando et al. 19 assimilated 17 studies on parental perspectives on caring for a child with OM. They found out that OM imposes a notable health burden on the child and can resemble a chronic disease. For parents residing in low middle income countries (LMIC) with poor socioeconomic status, it becomes even harder to make ends meet due to the expenses (direct and indirect) involved in treating this disease. In some cases, it may lead to catastrophic expenditure for the family. In such cases, the expenses of surgical treatment are not affordable for individuals of low-income strata.

## Review of Literature

### Search Strategy

**Fig. 1:**
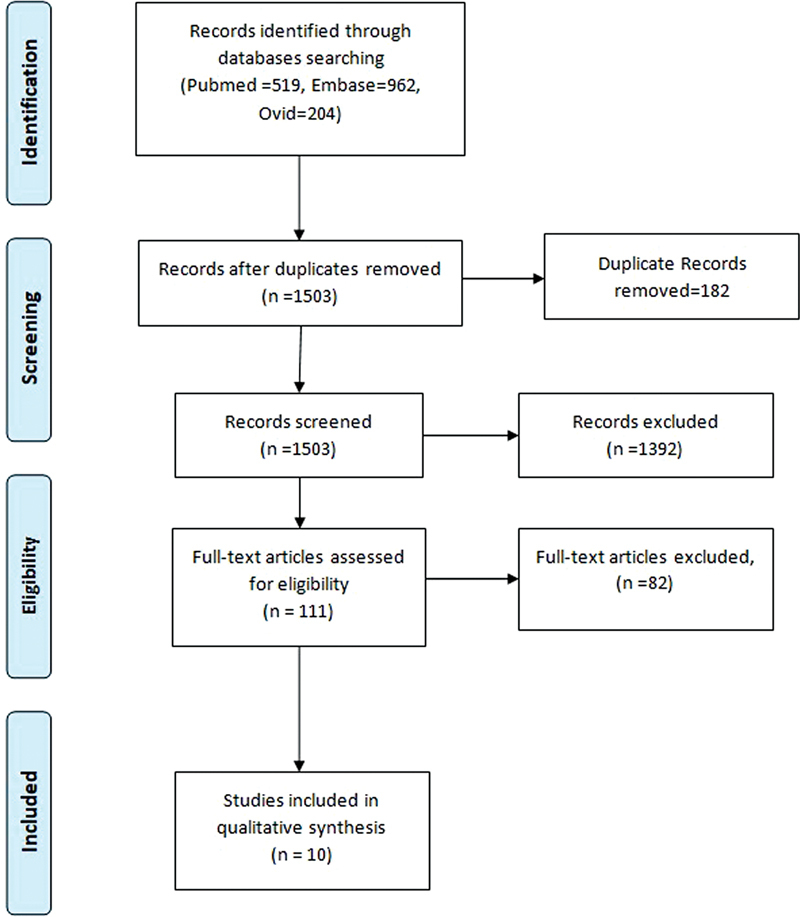
PRISMA Flowchart for literature review.

In the present review, the preferred reporting items for systematic reviews and meta-analysis (PRISMA) guidelines were followed. The literature search was done in PubMed Central, Ovid, and Embase to identify relevant studies. Full text articles were retrieved by two reviewers according to the inclusion and exclusion criteria. These articles were further reviewed by a third reviewer and conflicts were addressed. The PRISMA flowchart is given below ( Fig. 1 ) to depict the literature search.

### Inclusion and Exclusion Criteria

The cross-sectional studies that investigated economic burden associated with OM in children and adults were considered. All published articles since the year 2009 up to the year 2021 without restriction of language or geography were considered for the present review. The studies done before 2009 and those that did not give economic burden in monetary terms were excluded for the present review. The literature excluded were duplicate studies, commentaries, short communications, editorials, summaries, and letters.

### Methods for Data Extraction

A preformed data extraction format was utilized for the extraction of data from the studies included in the present systematic review. GDP deflator formulas were employed to adjust all costs to a specific price year (2022). This ensured that the effects of inflation are removed from the cost estimation. The results were represented in the form of tables.

To get optimum results, the GDP deflator formula was used to have cost estimation for a specific year (2022). 20

Conversion of nominal costs to real costs as per GDP deflator:





This gives the percentage change which is to be multiplied by the nominal cost.

## Results

The literature search led to the inclusion of ten studies according to the inclusion and exclusion criteria. These studies were finalized for data extraction enumerating the economic burden of OM for various countries in monetary terms. The table below ( Table 1 ) gives the characteristics of included studies and OM-related economic burden in various countries. The various instruments used for data collection were the Health Economic Questionnaire, the Productivity and Disease Questionnaire (PRODISQ), the Health and Labor Questionnaire (HLQ), and the Productivity Cost Questionnaire. Eight studies had children as subjects and two had mixed population. Direct costs (health system and patient perspective) ranged from USD 122.64 (Netherlands) to USD 633.6 (USA) per episode of OM. Looking at only the patient perspective, the costs ranged from USD 19.32 (Oman) to USD 80.5 (Saudi Arabia). The total costs (direct and indirect) ranged from USD 232.7 to USD 977 (UK) per episode of OM. The economic burden per year ranged from USD 73 million (Turkey) to USD 5 billion (USA).

**Table 1:** Studies included showing direct and total costs (including productivity loss and loss of wages) per episode of OM in USD for year 2022

Study ID	Country	Year of study	Methodology	Subjects/Sample size	Form of OM	Direct costs	Total costs	Economic burden per year
Speets et al. 30	Sweden	2009	Internet survey of parents of children < 5 years old	Children/91 MD confirmed episodes	AOM	315	751	87 million
Ahmed et al. 36	USA	2009	Data from Medical Expenditure Panel survey	Children/995	AOM	409 from healthcare system perspective		4 billion
Coronell-Rodríguez et al. 16	Colombia	2015	Questionnaires to parents	62 episodes of OM	AOM	166.4	232.7	
Mustafa et al *.* 25	Saudi Arabia, Oman, Pakistan, Turkey	2013	Data from one year Medical records of children	Children < 5 years old /4,043 children	AOM	Saudi Arabia- 80.5;Oman-19.32;Pakistan-26.5; Turkey-40.3 from patient perspective		
Dinleyici et al *.* 37	Turkey	2010	Data from national convenience sample	Children < 5 years old/600	AOM	36.4 from patient perspective		73 million
Wolleswinkel-van den Bosch et al *.* 10	Belgium, France, Germany, Italy, Netherlands, Spain, UK	2010	Internet questionnaire for the parents of children	Children < 5 years old/1,293 physician confirmed	AOM	Belgium-131.3;France-228.8;Germany-152;Italy-227.5;Netherlands-84.5;Spain-280;UK-218.4	Belgium-556;France-572;Germany-916;Italy-683;Netherlands-432;Spain-789;UK-977	
van Uum et al *.* 38	Netherlands	2018	Data in the form of questionnaires	Children < 5 years old/223	AOM	122.64	713.4	80 million
Crawford et al *.* 39	Malaysia	2014	Data in the form of questionnaires	Children ≤ 5 years old/110	AOM		116.4	
Kim et al *.* 12	Korea	2012	National Health Insurance Claims data	All age groups/1,788,303	AOM	310.6	361.2	
Tong et al *.* 27	USA	2014	Data from US insurance records in the Truven MarketScan database	All age groups	AOM	633.6 including emergency treatments		5 billion

Abbreviations: AOM, acute otitis media; OM, otitis media.

**Fig. 2:**
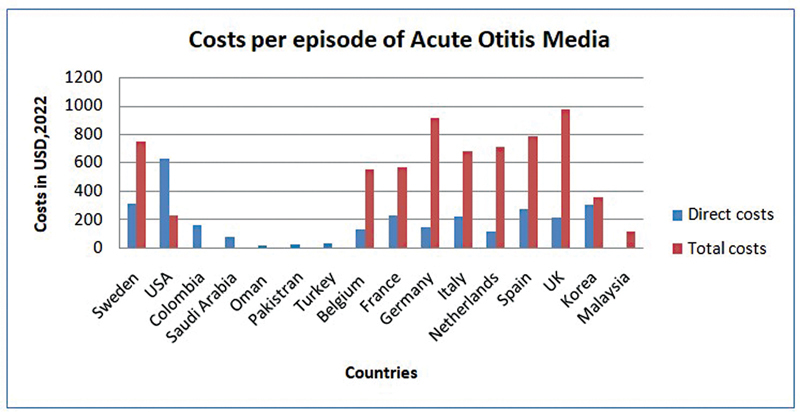
Graphical representation of direct and indirect costs in various parts of the world.

Direct and indirect costs per episode of AOM are depicted in the form of graph as shown below ( Fig. 2 ).

For the countries that appeared in more than one study, costs from the most recent study were included for analysis. European countries show high total costs since per capita income is high, which makes most part of indirect costs which includes loss of wages.

## Discussion

Studies conducted in different parts of the world showed that Pneumococcal Conjugate Vaccination (PCV) of children may plausibly reduce both the burden of AOM and its related cost. 21 22 The World Health Organization (WHO) in 2007 recommended the incorporation of pneumococcal vaccination in national immunization programs (NIPs). 23 However, a recent review published in the Lancet on the effectiveness of pneumococcal conjugate vaccine depicted that when administrating a vaccine containing various types of *pneumococcus* together with a carrier protein from bacterium called *Haemophilus influenzae* (PHiD-CV10), reduces the risk of experiencing acute middle ear infections, but these estimates did not reach significance. 24 The study done by Mustafa et al. in 2013 documented that PCV were included in NIP in Oman (PCV-7; 2008; 99% coverage), Saudi Arabia (PCV-7; 2009; 98% coverage), Turkey (PCV-7; 2008; 97% coverage), Pakistan (PHiD-CV; 2012; 66% coverage), UK (PHiD-CV; 66% coverage). During this study, the incidence of AOM was found to be higher among vaccinated population in Oman and Turkey in children. Saudi Arabia had a similar incidence and Pakistan saw a lower incidence in vaccinated subjects. 25 In the UK and in the USA, PCV-7 and PCV-10 were introduced in 2010 and it reduced incidence of AOM. 26 In Korea, PCV-7 and PCV-10 were introduced in NIP since 2014 and it reduced the incidence as well as the economic burden by 18 percent. But with the change in the pathology of OM causing pathogen, the effect of vaccination needs to be evaluated and now its increasing incidence in adults (between 50 and 59 years old) is of concern. 12 After the introduction of PCV-13 in the USA, its effect on AOM cases is unclear as *Streptococcus pneumonia* remained the cause of most AOM cases. Children < 5 years old had the highest rate of AOM and from 2008 to 2014 overall incidence rates changed little. Rates decreased for youngest children but increased for adult groups. 27

Although the PCVs are not currently included in the NIP in Malaysia, two PCVs are available in the private market: a 13-valent pneumococcal conjugate vaccine (PCV13; *Prevenar 13* ) and a pneumococcal polysaccharide and NTHi protein D conjugate vaccine (PHiD-CV; *Synflorix* ). In a cost-effectiveness study done in Malaysia, when compared with no vaccination, a PHiD-CV 2 + 1 program was projected to prevent 1,109 cases of invasive pneumococcal disease (IPD), 24,679 of pneumonia and 72,940 of AOM over 10 years, with additional costs of USD 30.9 million and 1084 QALYs, respectively, at an ICER of USD 28,497/QALY. 28 In another cost effectiveness study done in Korea, PHiD-CV (10-valent pneumococcal NTHi protein D conjugate vaccine) was projected to prevent an additional 195,262 cases of pneumococcal diseases and NTHi-related diseases versus PCV-13. Compared to PCV-13, PHiD-CV was projected to provide similar prevention against IPD and community-acquired pneumonia but would prevent more cases of AOM. 29

The results of our economic evaluation concluded that countries with strong economies having high economic costs as high wages here leads to higher indirect costs. In Sweden, the episodes of medically diagnosed OM for which caregivers had to stay home from a paid job was about 57%. Workdays that are lost from a paid or unpaid job majorly depends on the labor-force participation of parents, especially women. In Sweden, 63 percent of the women work full-time. Therefore, the indirect costs and opportunity costs were higher for Sweden. 30 In the study done in seven European countries, the hourly labor costs such as housekeeping also added to the direct costs. The direct costs in Italy, Spain, France, Germany, and UK were mainly due to the costs of hospitalization and visits to the physicians. Indirect costs associated with productivity losses were higher for the UK, as the hourly cost of labor is high and parents reported productivity loss while at work. In most of the studies, when OM was diagnosed, 85 to 97% of the patients were prescribed antibiotics. 10 This also added up to the economic burden. In countries where GP plays the gate keeping role, the visits are higher (Sweden-47%, UK-82%, Netherlands-87%). In countries where the role of family doctor is played by the pediatrician, the percentage of visits to GP were lower. 10 The prospective observational study done in middle eastern countries with the sample size of 4,043 (2,750 children had received ≥ 1 dose of PCV), assessed economic burden from the parent/caregiver perspective using the Health Economic questionnaire. These countries (Turkey, Saudi Arabia, Pakistan, Oman) showed low direct costs compared with the European countries. The children of most of the countries, except Pakistan, had medical insurance or were covered by public healthcare facility. 24 In developing countries, direct costs exceeded indirect costs. Direct costs are low in countries where the healthcare expenses are borne by the national healthcare systems. More studies in developing countries are required to look into economic burden of OM from the societal perspective.

## Disease Burden in the Form of Disability Adjusted Life Years

One disability adjusted life year (DALY) represents the loss of the equivalent of one year of full health. Disability adjusted life years for a disease or health condition are the sum of the years of life lost to due to premature mortality (YLLs) and the years lived with a disability (YLDs) due to prevalent cases of the disease or health condition in a population. The loss of function is based on the frontier national life expectancy projected for the year 2050 by the World Population Prospects 2012 (UN Population Division, 2013), with a life expectancy at birth of 92 years. Prevalence YLDs are used here. Prevalence of YLDs are calculated as the prevalence of each nonfatal condition multiplied by its disability weight. 31

DALY = Mortality (YLLs) *Morbidity (YLDs) Disutility weight of OM = 0.005 32

If life expectancy is 92 years and a patient dies at the age of 82 due to OM, 10 years of life are lost (YLLs). If a person lives with the disease for 10 years (YLDs), morbidity caused is 10* 0.005.

DALY = 10*0.05= 0.50 in case of 10 years of life lost and 10 years of life lived with disability. Otitis media accounted for 1,806,500 disability-adjusted life years (DALY globally) during 2013. Otitis media-related DALYs in Korea decreased from 19.86 per 100,000 in 1900 to 17.02 per 100,000 in 2013. Disability adjusted life years can be averted with new diagnostics, prevention, or treatment interventions. Therefore, cost effectiveness of any intervention can be given in terms of cost per DALY averted.

## Effectiveness of Interventions in the Form of Quality Adjusted Life Years

The quality adjusted life years (QALYs) is a measure of the value of health outcomes. Since health is a summation of length of life and quality of life, the QALY was developed as an attempt to combine the value of these health attributes. The QALY is calculated by considering the change in utility value induced by the treatment which is multiplied by the duration of the treatment effect to provide the number of QALYs gained. Quality adjusted life years can then be incorporated with medical costs to arrive at a final common denominator of cost/QALY. This parameter can be used to compare the cost-effectiveness of any treatment. QALY assumes that a year of life lived in perfect health is worth 1 QALY and that a year of life lived in a state of less than this perfect health is worth < 1. In order to determine the exact QALY value, it is sufficient to multiply the utility value associated with a given state of health by the years lived in that state. Half a year lived in perfect health is equivalent to 0.5 QALYs (0.5 years × 1 Utility), the same as 1 year of life lived in a situation with utility 0.5 (quality of life with otitis media) * (1 year × 0.5 Utility). 33

## Final comments

Otitis media undoubtedly poses an immense burden due to high economic costs and low QoL of children and their caregivers. Early detection and prompt treatment would be the right approach. Economic burden was found to be high in the developed nations. High direct costs in developing nations resulted in high out of pocket expenditures due to OM episodes. High indirect costs in the developed nations increased the economic burden exponentially. Future research should assess motivators for prescribing and evaluating patient outcomes among clinicians for the process of decision-making. 34 In most countries, antibiotics is the first line of treatment, but antibiotic resistance is on the rise globally; therefore, other approaches as ‘watch-and-wait’, prevention through vaccination or incorporation of better diagnostic techniques should be considered. Watchful waiting (without prescribing antibiotics) for AOM was proved to be cost effective in a study done in the United Nations in the year 2017. 35
